# Predictors of High-School Dropout Among Ultraorthodox Jewish Youth

**DOI:** 10.3389/fpsyg.2020.01911

**Published:** 2020-08-11

**Authors:** Yael Itzhaki-Braun, Haya Itzhaky, Yaacov B. Yablon

**Affiliations:** ^1^The Gershon Gordon Faculty of Social Sciences, The Bob Shapell School of Social Work, Tel Aviv University, Tel Aviv, Israel; ^2^Faculty of Social Sciences, School of Social Work, Bar-Ilan University, Ramat Gan, Israel; ^3^Faculty of Social Sciences, School of Education, Bar-Ilan University, Ramat Gan, Israel

**Keywords:** high school dropout, the Ultraorthodox Jewish community, parental conditional regard, societal conditional regard, newly religious family

## Abstract

Focusing on the unique social characteristics of closed communities, the current study examined the predictors of high-school dropout among Ultraorthodox Jewish youth, focusing on background variables [i.e., individual’s age, family’s religious group affiliation, and other high-school dropout(s) in family]; social resources (i.e., self-esteem and mastery); and parental conditional regard (PCR) and societal conditional regard (SCR), with reference to both positive regard and negative regard. The study was conducted in Israel with the participation of 261 Ultraorthodox Jewish males, ages 14–21 (*M* = 17, *SD* = 1.17), who were at different stages in the dropout process. Path analysis modeling indicated that being a member of a newly religious family, or of a family in which another member had already dropped out of school, was a predictor of dropout. Surprisingly, personal resources were not found to be a predictor of dropout, whereas parental conditional regard and societal conditional negative regard (SCNR) were found to be the most significant predictors. Findings highlight the unique predictors of high-school dropout among youth from the Ultraorthodox Jewish community, and the role of PCR and SCR in this process.

## Introduction

Dropping out of high school is a risk situation for youth, with negative personal and social consequences, as discussed in the research literature at length (Rumberger and Lim, 2008; Staff and Kreager, 2008; Makarova and Birman, 2015). Thus, many studies have been conducted in order to identify the predictors of high-school dropout among youth at risk (Janosz et al., 2008; Archambault et al., 2009), under the assumption that recognizing at-risk youth would be helpful in the prevention of their dropping out (Ricard and Pelletier, 2016). Yet, there is very little research or specific data, if any, regarding the predictors of high-school dropout among youth from closed religious communities, despite the fact that this phenomenon, and the percentage of students from these communities in society at large, is growing (Israeli Central Bureau of Statistics, 2016). Given that in these communities, dropping out of school reflects a violation of community norms (Kelly, 2014), the underlying rationale of this research was the expectation that there would be unique predictors for high-school dropout among youth in this population. As such, the aim of the current study was to identify possible predictors of high-school dropout in a closed religious community. Specifically, we focused on three groups of students from the Ultraorthodox Jewish community in Israel, who represent three different stages in the dropout process. These three groups were defined as (1) students who were at risk of dropping out of high school, (2) students who had dropped out of high school and enrolled in a program for dropouts, and (3) students who had dropped out of school and did not enrolled in any educational framework at all. Based on social capital theory (Coleman, 1988), we used an ecological perspective and investigated the contribution of background variables, personal resources, family resources, and communal resources, to high-school dropout in this community.

### Dropping Out of School in Religious Communities

Youth who dropped out from school can engage in many risk situations. These include unemployment, poverty, health issues, early death, and crime (Belfield and Levin, 2007; Brekke, 2014). Young people who drop out of high school are therefore considered to be an at-risk group. For youths from religious communities, the repercussions of dropout are even greater, as the individuals in these communities live in accordance with strict norms. For instance, individuals are expected to study in the community’s schools, which “multitask:” that is, they educate, provide a framework for socializing, and indoctrinate. However, when individuals leave this framework, they effectively reject communal norms, potentially distancing themselves from their communities (Finkelman, 2011; Cates and Weber, 2013). Conflicts between the youths and their surrounded systems, the parents, and the community (Yogev, 2012) are therefore created when a young person drops out of school in these settings.

The current study focused on Israel’s Ultraorthodox Jewish sector, a sector that constitutes 9% of Israel’s overall Jewish population (Israeli Central Bureau of Statistics, 2019). These individuals live in closed-off neighborhoods (away from secular neighborhoods), have specified dress codes for men and women, and strictly observe Jewish law. The youth of these communities attend state-certified private educational institutions, with content uniquely intended for them. The schools are single-sex. In high school, boys focus on Jewish subjects at their “yeshivas” and continue studying in this manner until marriage (Finkelman, 2011). The yeshiva high school plays many roles – religious, educational, social – and its goal is to mold the boys’ behavior to be in line with the tenets of Ultraorthodox Judaism. Ultraorthodox Jewish parents send their sons to yeshiva high schools for reasons similar to those of parents who send their children to other kinds of private religious schools (e.g., Christian, Mormon, Amish): to maintain their children’s observance of the religion, faith to community values, and distance from the secular world (Irwin, 2002; Cates and Weber, 2013; Bertrand, 2015).

Surprisingly, there is a relatively high rate of high-school dropout in the Ultraorthodox Jewish community as compared with the rate of high-school dropout in the state education framework (Israeli Central Bureau of Statistics, 2018), making the topic one of growing interest and concern. However, there has been almost no research conducted in regard to the predictors of high-school dropout in this community. The large role that the yeshiva plays in the youths’ lives, and the consequences of leaving this framework (as described above), requires a broad perspective that takes into account the familial and communal aspects that typify this community. Based on other studies that have investigated high-school dropout, this study too was based on social capital theory (Perreira et al., 2006; South et al., 2007).

### Social Capital Theory

As stated above, the theory of social capital (Coleman, 1988) provided the basis for the current study, because it enables a theoretical base to the predictors of dropping out of high school in a closed religious community. Coleman (1988) was the first to provide evidence of a relationship between social capital and school dropout rates. He argued that students attending private religious schools were less likely to drop out than students attending public high schools due to the relationships of parents with one another, in the schools, and in their community (Coleman and Hoffer, 1987). Social capital theory focuses on different levels of factors surrounding the individual: human capital and social capital. Human capital refers to individuals’ skills, capabilities, and resources that make them able to act in new ways (Coleman, 1988). Social capital refers to the value of social structures that act as resources that individuals can use to fulfill their needs. The most common structure is the family, while other conceptualizations of social capital contain various other sources, like school, neighborhood, and community, broader familial forms of social capital (Coleman, 1988; Long and Perkins, 2007; Dufur et al., 2008; Goksen and Cemalcilar, 2010; Hassan et al., 2016). Social capital theory emphasizes the importance of the individual’s personal resources, his community resources, and the interaction between the two, as well as the researcher’s ability to use them to predict, among other things, school dropout among youth (Perreira et al., 2006; South et al., 2007).

High levels of solidarity among members and few social connections they have with people outside their communities in closed religious communities may be the factor for the higher levels of social capital that were found in these communities compared to non-religious communities (Coleman, 1988; Portes, 1998; Fukuyama, 2001). It was found that religious participation alters teen social networks, putting teens in greater contact with educational resources and pro-school values from peers, all relating to the lower percentage of drop out compare to non-religious youth (Glanville et al., 2008). This may result from the ability of congregations to provide a place to meet and develop friendships with non-at-risk youth. Regarding youth from the Ultraorthodox Jewish community, the loss of social capital is considered to be a significant issue in their school dropout process (Kelly, 2014).

In a review of 203 published articles, Rumberger and Lim (2008) found that high-school dropout predictors were divided into two main groups: individual factors including background variables and self-perception, and institutional factors including family and community resources. In the current study, we also investigated background variables, and personal and social resources, as predictors of dropout among Ultraorthodox Jewish youth. We chose to focus on background variables that have been found to be relevant in predicting dropout in the literature in regard to this particular population. Specifically, we investigated the contribution of age, being a member of a newly religious family and/or of a family in which another family member had already dropped out of school. Regarding personal resources, we investigated the contribution of self-esteem and mastery. Regarding social resources, we focused both on the family aspect, via parental conditional regard (PCR) (Assor et al., 2004), and on the community aspect, via societal conditional regard (SCR) (Itzhaki et al., 2018).

### Background Variables

Rumberger and Lim’s meta-analysis (2008) concluded that 31 of the 57 studies that investigated the relationship between age and dropout found that older high school students had a higher chance to drop out and a low probability to graduate, than younger high school students (Chapman et al., 2010; Landis and Reschly, 2011). Moreover, dropout could happen in lockstep with the decision of a sibling to drop out (Dupéré et al., 2015). Consistently, high school students were more likely to drop out if they had a sibling who had done so (Rumberger and Thomas, 2000; Jacob, 2001).

A concept that seems to be somewhat unique to the Ultraorthodox Jewish community is that of the “newly religious family.” The term newly religious family (“hozrim b’tshuvah”) refers to those who “return” (even if they were not brought up as such) to Orthodox Jewish religious observance (Davidman and Greil, 2007). This group comprises people who for the most part had never observed Jewish religious law until they decided to become part of the Jewish religious community. In most cases, the community chosen was the Ultraorthodox Jewish community. Although the “hazarah b’tshuva” process is generally seen as being praiseworthy by religious Jews, newly religious families rarely succeed in fully integrating into the Ultraorthodox Jewish community, mostly because Ultraorthodox communities are quite closed and wary of assimilation (Doron, 2013). Perhaps for this reason, the percentage of high-school dropout among newly religious families (a minority group of the Ultraorthodox Jewish community) is higher than that of “established” Ultraorthodox Jewish families (Itzhaki, 2009; Yogev, 2012).

### Self-Esteem and Mastery

In this study, similar to previous studies that investigated youth, the personal resources that were examined are self-esteem and sense of mastery (Lipschitz-Elhawi and Itzhaky, 2005; Phillips, 2010; Dwyer et al., 2011). Unsurprisingly, youth at risk are characterized by low levels of self-esteem and low levels of mastery, among other things (Jessor et al., 1998; Metz, 2006), both of which are connected to a failure to adapt into frameworks such as school or the workplace (Dillon, 2004; Aristilde, 2006). Both self-esteem and mastery were found to be predictors of high-school dropout (Bong and Skaalvik, 2003; De Ridder et al., 2013; Quiroga et al., 2013).

### The Familial and Communal Aspect

Both family and community are described in the literature as crucial factors in predicting school dropout among youth. The familial aspect has been investigated via three main components: (1) family structure, (2) family resources, and (3) family practices (Rumberger and Lim, 2008). The relationship between adolescence and parents was found to have a crucial role in school engagement, while poor parent–teenager relationships were found to be a predictor for high-school dropout (Roberts et al., 2013; Fortin et al., 2013; Wang and Fredricks, 2014). As for the community aspect, it has been investigated via the following: (1) access to institutional resources (e.g., childcare, medical facilities, employment opportunities), (2) parental relationships that can provide access to family and friends, as well as social connections in the neighborhood, and (3) social capital that arises out of mutual trust and shared values and that comes into play in terms of supervising and monitoring the activities of residents, and particularly youth (Leventhal and Brooks-Gunn, 2000). High level of trust and mutual cooperation among people in the community contributed to reducing high-school dropout (Hassan et al., 2016). However, negative attitude from community members such as discrimination and prejudice was found to be a predictor of high-school dropout (De Witte et al., 2013). In the current study, we focused on the examination of the role of family and community via the relationships between the youths and their parents and between the youths and the members of their community. Specifically, these relationships were examined via the construct of conditional regard (CR).

### Parental Conditional Regard and Societal Conditional Regard

In the current study, we examined the contribution of parental conditional regard (PCR), and societal conditional regard (SCR) (Itzhaki et al., 2018), to school dropout among youth. Parental conditional regard has its basis in self-determination theory (SDT) (Ryan and Deci, 2000) and is a socialization strategy wherein parental love/acceptance depends upon the child’s complying with the parents’ expectations. Parental conditional *positive* regard (PCPR) implies a greater degree of parental attention, affection, and esteem as a result of the child meeting parental expectations. Parental conditional *negative* regard (PCNR), conversely, implies a lesser degree of parental affection/warmth as a result of the child *not* meeting parental expectations (Assor et al., 2004). What the constructs of PCPR and PCNR have in common is that both hinder autonomy and give rise to a number of negative effects (Assor et al., 2004; Roth et al., 2009; Assor and Tal, 2012).

The authors, in an earlier study (Itzhaki et al., 2018), argued that in closed traditional societies, CR as practiced by the society could affect the individual in much the same way that CR, as practiced by the parents, would affect him/her. As such, one needs to understand societal conditional regard (SCR) in order to understand individuals’ behaviors in closed religious communities. Societal conditional regard, an adaptation of PCR, refers to the way in which *society* “gives its blessing” to individuals on the basis of their realizing what that particular society expects of them. In a set of studies regarding high-school dropout in the Ultraorthodox Jewish community, the authors found that both PCR and SCR contributed to psychological aspects in the youths’ lives.

Both PCNR and PCPR were found to contribute to higher levels of loneliness and to lower levels of well-being among high-school dropouts. Societal conditional negative regard (SCNR) made a negative contribution to psychological aspects, i.e., higher levels of SCNR contributed to higher levels of loneliness and lower levels of well-being. However, societal conditional positive regard (SCPR) was found to contribute to higher levels of positive future orientation and well-being (Itzhaki et al., 2018). Following these novel findings, in the current study, the authors wished to find out whether PCR and SCR might also serve as predictors of high-school dropout among youth from the Ultraorthodox Jewish community, beyond their psychological consequences. According to SDT, less autonomy practices relate to less self-determined motivation among youth, which in turn predicts high-school dropout (Vallerand et al., 1997; Hardre and Reeve, 2003). Therefore, the authors examined PCR and SCR in regard to youths who no longer practiced a religious lifestyle that complied with Jewish law (Assor and Friedman, 2005) and hypothesized that higher levels of conditional regard (CR) would predict their school dropout, as a result of their insecure relationships with both their parents and their community.

In sum, the goal of this study was to examine the contribution of background variables, personal resources, and social resources to the phenomenon of high-school dropout among Ultraorthodox Jewish youth in Israel. To do so, three groups of students who were at three different stages of the dropout process were examined. Based on social capital theory (Coleman, 1988), the hypothesis was that older age, belonging to a newly religious family, having a sibling who had dropped out of school previously, having low levels of self-esteem and mastery, and being the recipients of high levels of CR would be predictors of belonging to the dropout group (in comparison to belonging to the other two study groups). By comparing three groups of students representing three distinct levels in the dropout process (i.e., at risk of dropping out; dropping out but participating in alternative educational programs; and dropping out but not participating in any other educational framework), the conditions were set for a deeper inquiry into the contribution of each of the studied predictors to the phenomenon of high-school dropout in a closed religious community.

## Materials and Methods

### Participants and Procedure

Participants comprised 261 Jewish male youths who were born and raised in an Ultraorthodox family and originally were part of the Ultraorthodox community between the ages of 14 and 21. Participants were divided into three research groups representing three different stages in the dropout process. One group consisted of youths in yeshiva high schools who were at risk for dropping out. These youths assigned or received treatment from the social services department designated for yeshiva boys (*n* = 61). A second group consisted of youths who had dropped out of yeshiva and enrolled in a program designed especially for Ultraorthodox high-school dropouts (*n* = 131), and a third group consisted of youths who dropped out of high school and did not enroll in any educational framework (*n* = 69). All participants were members of the same community located in the center of Israel, and they shared the same community background. The average age of the participants was 17 (*SD* = 1.71). Most of the participants (67%) defined themselves, religiously, as Ultraorthodox (or “Haredi”). Some of the participants (19.6%) defined themselves as “not religious,” and the rest of them (13.4%) defined themselves as “religious.”

Following the approval of data collection and questionnaires by Bar-Ilan University’s institutional review board (IRB), a convenience sample was obtained, and participants were recruited by a variety of youth practitioners who meet the youth in the different services according to the inclusion criteria: male, singles, who originally were part of the Ultraorthodox community, between the ages of 14 and 21. Participants for the yeshiva high school group were recruited by practitioners from the mentoring organizations and from the social services department designated for yeshiva boys. Participants for the program for high-school dropouts were recruited by practitioners who worked in these programs. Participants for the dropout group were recruited by practitioners who worked with dropout Ultraorthodox youth in the street. The practitioners administrate the questionnaires to participants during their meetings. In order to assure anonymity of the respondents, the participants were told not to write their names or provide any identifying information on the forms. Participants’ agreement was voluntary, and participants were told that they were free to stop answering questions whenever they wished to. Of the ∼400 questionnaires that were distributed, 276 of them (or ∼69%) were returned, and 15 of them (about 3.75%) were eliminated due to technical problems, such as partial completion.

### Instruments

#### Self-Esteem

This questionnaire was developed by Rosenberg (1965) and assesses the individual’s sense of self-esteem. It consists of 10 items such as the following: “I feel that I’m a person of worth, at least on an equal plane with others.” The participant in the current study was asked to rate the extent to which he agreed with each item on a 5-point Likert-type scale, ranging from 1 *(strongly disagree)* to 5 *(strongly agree)*. The Cronbach’s alpha reliability of the questionnaire in the present study for this measure was α = 0.77.

#### Mastery

This questionnaire was developed by Pearlin and Schooler (1978) and assesses an individual’s sense of mastery over his surroundings and over the future. It consists of seven items such as “The future and what will happen to me depends mostly on me.” The participant is asked to rate the extent to which he/she agrees with each item on a 5-point Likert-type scale, ranging from *strongly disagree* to *strongly agree*. The Cronbach’s alpha reliability reported by Pearlin and Schooler was α = 0.88, and the reliability of the questionnaire used in the present study was α = 0.78.

#### Parental Conditional Regard

This questionnaire was based on the parental conditional regard (PCR) questionnaire originally developed by Assor et al. (2004). It assesses the degree of parents’ conditional regard, as experienced by the youth, and also assesses the youth’s perceptions of positive and negative parental conditional regard. In addition, like the research of Assor and Friedman (2005), the current research also examined PCR in relation to the individual’s observance of a religious lifestyle and adherence to Jewish law. The questionnaire therefore consisted of 10 items. Results of an exploratory factor analysis yielded the expected two-factor structure with no cross loadings. Five of those items related to parental conditional positive regard (PCPR), such as “When I keep the commandments, I feel that my parents give me more warmth and affection than usual.” The other five items addressed parental conditional negative regard (PCNR), such as “When I do not immerse myself in my studies, my parents give me the feeling that I am not worthy.” The participant was asked to evaluate his agreement on a 5-point Likert-type scale ranging from 1 (*greatly agree)* to 5 (*do not agree at all)*. The Cronbach’s alpha reliability in the present study was.85 for PCPR and.94 for PCNR.

#### Societal Conditional Regard

This questionnaire was developed by Itzhaki et al. (2018) and was based on the parental conditional regard (PCR) questionnaire (Assor et al., 2004), which has already been described in detail above. While the PCR questionnaire used in this study was adapted with questions regarding observance of a religious lifestyle, the societal conditional regard (SCR) questionnaire was further adapted by replacing the word “parents” with the words “people in the community.” The SCR items were the same as the PCR items, except “my parents” was replaced by “people in the community” (e.g., “If or when I am careful about keeping the commandments, people in the community show me more warmth and affection than usual”). The Cronbach’s alpha reliability of the questionnaire used in the present study was.96 for societal conditional positive regard (SCPR) and.95 for societal conditional negative regard (SCNR).

#### Sociodemographics

This questionnaire was used to examine sociodemographic characteristics such as age, place of residence, and educational framework (or lack thereof). In this study, participants were also asked whether their family was newly religious (“*hozrim b’tshuva*”) or whether they had always been Ultraorthodox and whether they had a family member who had previously dropped out of high school.

## Results

### Correlations and Regressions

Correlations, means, and standard deviations among the study’s variables are presented in Table 1. As can be seen, there are significant correlations between some of the variables, without multicollinearity.

**TABLE 1:** Means, standard deviations, and correlations between the study variables.

Measures	*M*	*SD*	1	2	3	4	5	6	7	8	9
1.Age	17.05	1.71	–	–0.10	–0.02	–0.02	–0.01	–0.06	0.07	–0.07	–0.03
2.Newly religious family	0.48	0.50		–	0.20**	0.19**	0.20**	0.01	−0.13*	−0.21***	–0.11
3.Dropout family member	0.39	0.49			–	–0.11	–0.09	0.03	0.10	0.02	0.02
4.Self-esteem	3.77	0.72				–	0.63***	0.06	−0.44***	−0.16**	−0.31***
5.Mastery	3.56	0.89					–	–0.00	−0.44***	−0.16*	−0.31***
6.PCPR	4.19	0.96						–	0.26***	0.31***	0.25***
7.PCNR	2.48	1.37							–	0.38***	0.61***
8.SCPR	3.08	1.53								–	0.59***
9.SCNR	2.55	1.42									–

*p < 0.05, **p < 0.01, ***p < 0.001.

In order to examine the effects of the study’s variables as predictors of membership in each study group, we conducted four nominal regression models, where membership in a study group served as the dependent variable in two dummy categories: high-school students vs. dropouts, and youth in a program for dropouts vs. dropouts in no program. In the first model, we examined the contribution of age, newly religious family, and dropout family member. In the second model, we added the personal resources self-esteem and mastery. In the third model, we added the PCPR and the PCNR, and in the last model we added the SCPR and the SCNR. The regression coefficients for high-school students vs. dropouts are displayed in Table 2. In the first model, having a newly religious family and having a family member who had previously dropped out of school contributed to belonging to the dropout group. In the second model, self-esteem and mastery made no contribution to the group belonging. In the third model, PCR made no contribution to the group belonging. In the final model, entering the SCR, both PCPR and SCNR contributed to belonging to the dropout group. The regression coefficients and correlations for students in a program for dropouts vs. dropouts in no program are displayed in Table 3. In the first model, older age contributed to belonging to the dropout group, while having a newly religious family and having a family member who had previously dropped out of school made no significant contribution to group belonging. In the second model, self-esteem and mastery made no contribution to the group belonging. In the third model, PCR made significant contribution to the belonging to the dropout group. In the final model, entering the SCR, PCPR, and SCNR contributed to the belonging to the dropout group. The variables explained 38% of the variance in each of the two models.

**TABLE 2:** Predictors for high-school dropout among high school students vs. dropouts in the multi-nominal regression models.

	Model I			Model II			Model III			Model IV		
Measures	B (SE)	Wald (95% CI)	OR	B (SE)	Wald (95% CI)	OR	B (SE)	Wald (95% CI)	OR	B (SE)	Wald (95% CI)	OR
Age	0.05 (0.11)	0.22 (0.85–1.31)	1.05	0.04 (0.11)	0.16 (0.84–1.30)	1.04	0.01 (0.12)	0.01 (0.81–1.23)	1.01	−0.00 (0.12)	0.00 (0.79–1.26)	1.00
Newly religious family	−0.75 (0.37)	4.02* (0.22–0.98)	0.47	−0.90 (0.39)	5.35* (0.19–0.87)	0.41	−1.00 (0.41)	5.77* (0.17–0.84)	0.38	−0.94 (0.42)	5.05* (0.17-0.89)	0.39
Dropout family member	−1.00 (0.038)	7.07** (0.18–0.77)	0.37	−0.90 (0.38)	5.67* (0.19–0.85)	0.40	−0.88 (0.39)	5.03* (0.19–0.89)	0.41	−0.88 (0.40)	4.82* (0.19–0.91)	0.41
Self-esteem	–	–	–	0.06 (0.32)	0.03 (0.56–2.00)	1.06	−0.12 (0.36)	0.11 (0.44–1.80)	0.89	−0.16 (0.37)	0.19 (0.42–1.75)	0.85
Mastery	–	–	–	0.4 (0.27)	2.40 (0.89–2.54)	1.50	0.24 (0.29)	0.69 (0.72–2.22)	1.27	0.20 (0.29)	0.48 (0.69–2.17)	1.22
PCPR	–	–	–	–	–	–	−0.43 (0.24)	3.24 (0.40–1.04)	0.65	−0.52 (0.26)	3.87* (0.35–1.00)	0.59
PCNR	–	–	–	–	–	–	−0.48 (0.17)	8.17 (0.44–0.86)	0.62	−0.33 (0.19)	2.95 (0.49–1.05)	0.72
SCPR	–	–	–	–	–	–	–	–	–	0.28 (0.17)	2.82 (0.95–1.86)	1.33
SCNR	–	–	–	–	–	–	–	–	–	−0.47 (0.19)	5.89* (0.43–0.91)	0.62
*R*^2^		0.21***			0.24***			0.33***			0.38***	

*p < 0.05, **p < 0.01, ***p < 0.001.

**TABLE 3:** Predictors for high-school dropout stages among youth in a program for dropouts vs. dropouts in the multi-nominal regression model.

	Model I			Model II			Model III			Model IV		
Measures	B (SE)	Wald (95% CI)	OR	B (SE)	Wald (95% CI)	OR	B (SE)	Wald (95% CI)	OR	B (SE)	Wald (95% CI)	OR
Age	−0.40 (0.10)	17.33*** (0.55–0.81)	0.67	−0.40 (0.10)	16.85*** (0.55–0.81)	0.67	−0.43 (0.10)	16.48*** (0.53–0.80)	0.65	−0.46 (0.11)	17.75*** (0.51–0.78)	0.63
Newly religious family	0.36 (0.31)	1.39 (0.78–2.64)	1.44	0.17 (0.32)	0.27 (0.63–2.24)	1.19	0.08 (0.34)	0.06 (0.55–2.14)	1.08	0.09 (0.36)	0.07 (0.54–2.24)	1.09
Dropout family member	−0.18 (0.30)	0.36 (0.46–1.51)	0.83	−0.06 (0.31)	0.04 (0.51–1.73)	0.94	−0.02 (0.33)	0.00 (0.51–1.87)	0.98	−0.05 (0.34)	0.02 (0.49–1.87)	0.95
Self-esteem	–	–	–	0.37 (0.28)	1.75 (0.83–2.53)	1.45	0.23 (0.32)	0.54 (0.68–2.35)	1.36	0.19 (0.33)	0.33 (0.64–2.29)	1.21
Mastery	–	–	–	0.21 (0.23)	0.85 (0.79–1.94)	1.24	0.05 (0.25)	0.04 (0.65–1.70)	1.05	0.02 (0.26)	0.00 (0.61–1.69)	1.02
PCPR	–	–	–	–	–	–	−0.53 (0.22)	5.79* (0.38–0.91)	0.59	−0.57 (0.24)	5.44* (0.35–0.91)	0.56
PCNR	–	–	–	–	–	–	−0.45 (0.14)	9.79** (0.48–0.85)	0.64	−0.18 (0.17)	1.17 (0.60–1.16)	0.83
SCPR	–	–	–	–	–	–	–	–	–	0.20 (0.15)	1.81 (0.91–1.65)	1.23
SCNR	–	–	–	–	–	–	–	–	–	−0.62 (0.18)	12.45*** (0.38–0.76)	0.54
*R*^2^	0.21***			0.24***			0.33***			0.38***		

*p < 0.05, **p < 0.01, ***p < 0.001.

### Mediation Model

A path analysis model was performed using Mplus 8 (Muthén and Muthén, 1998–2017) to examine the possibility of a mediating model. In the model, the target variables of the model (i.e., high school students vs. dropouts, and students in program for dropouts vs. dropouts) were expected to respond to the study variables. Based on the regression findings, we entered into the model only the variables that made significant contributions to the regression models (i.e., older age, being from a newly religious family, having a family member who had previously dropped out of school, PCR, and SCR). In our path analysis model, we estimated indirect effects from the study’s background variables (i.e., older age, being from a newly religious family, and having a family member who had previously dropped out of school) to outcome variables (i.e., the three study groups) through mediators such as PCPR, PCNR, SCPR, and SCNR. All possible paths from independent variables to dependent variables were estimated.

**FIGURE 1:**
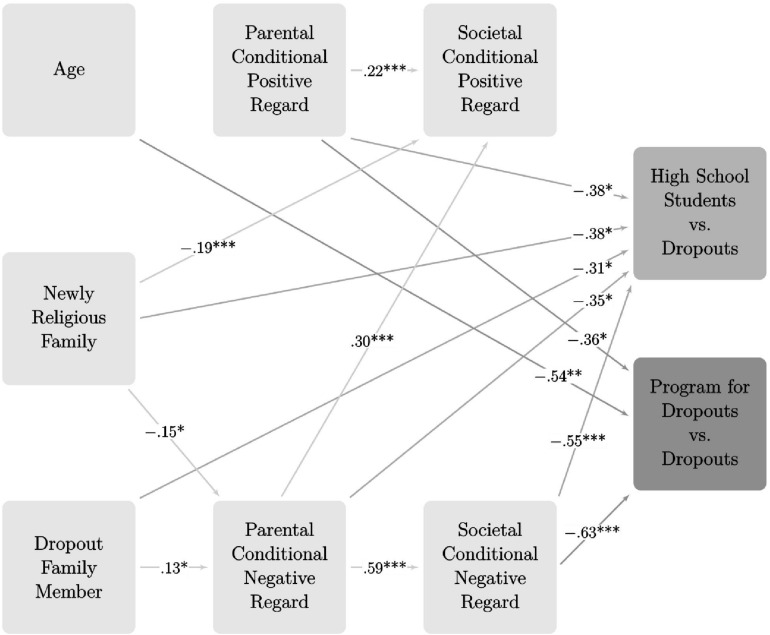
Standardized results of the path anlysis model of socio-demographic variables, PCR and SCR on stages in the dropout process.

Figure 1 presents the standardized β coefficients and SEs of the direct effects found to be significant in the path analysis modeling, *p* < 0.05 and lower (AIC = 5502, BIC = 5677). Table 4 presents the β coefficients and SEs of the indirect effects in the path analysis modeling.

**TABLE 4:** Betas and SEs of the indirect effects of the path analysis model.

Independent	Mediator	Dependent	Independent to mediator	Mediator to dependent	Direct effect	Indirect effect	95% CI
Newly religious family	PCNR	SCPR	−0.40* (0.17)	0.34*** (0.06)	−0.19*** (0.03)	−0.04* (0.02)	(−0.258, −0.014)
Newly religious parents	PCNR	SCNR	−0.40* (0.17)	0.61*** (0.05)	−0.03 (0.05)	−0.09* (0.04)	(−0.451, −0.040)
Dropout family member	PCNR	SCNR	0.37 (0.18)	0.61*** (0.05)	−0.03 (0.05)	(0.04)	(0.010, 0.440)
PCNR	SCNR	High school students vs. Dropouts	0.61*** (0.05)	−0.48** (0.17)	−0.35* (0.16)	−0.30** (0.11)	(−0.513, −0.085)
PCNR	SCNR	Program for dropouts vs. Dropouts	0.61*** (0.05)	−0.62*** (0.18)	−0.21 (0.15)	−0.38*** (0.12)	(−0.612, −0.159)

*p < 0.05, **p < 0.01, ***p < 0.001.

Regarding the connection between the study’s variables and “high school students vs. dropouts” group, the findings point to a direct negative correlation between having a newly religious family and having a family member who had previously dropped out of school and between the group. It was found that high school students were less likely to be part of newly religious families (β = −0.38) or to have family members who had previously dropped out of school (β = −0.31) than were dropouts.

A direct negative correlation was also found between PCPR, PCNR, and SCNR and between the groups. High school students experienced less PCPR (β = −0.38), less PCNR (β = −0.35), and less SCNR (β = −0.55), than did dropouts. Parental conditional negative regard also made an indirect contribution to the group via SCNR (β = −0.30).

Regarding the connection between the study’s variables and the “students in a program for dropouts vs. dropouts” group, the findings point to a direct negative correlation between age and group. It was found that students in a program for dropouts were older than dropouts who were in no educational framework (β = −0.54).

A direct negative correlation was also found between PCPR and SCNR and between the groups. Students in a program for dropouts experienced less PCPR (β = −0.36) and less SCNR (β = −0.63) than did dropouts who were in no educational framework. Parental conditional negative regard also made an indirect contribution to the group via SCNR (β = −*0.38)*.

## Discussion

The current study’s findings reveal unique predictive factors of high-school dropout among youth from an Ultraorthodox Jewish community in Israel. Self-esteem, and mastery, which are generally considered to be significant predictors for school dropout among youth, made no significant contribution to school dropout in the current study. Conditional regard (CR), on the other hand, a construct examined for the first time as a predictor of youth dropout, was indeed found to be a significant predictor, both in terms of parental CR and societal CR.

Consistent with previous studies, both “having a sibling who dropped out of school” and “belonging to a newly religious family” were found to be predictors of high-school dropout (Rumberger and Thomas, 2000; Itzhaki, 2009; Yogev, 2012). A sibling who drops out of yeshiva may serve as an inspiration for a brother who feels he does not “fit in” or who wants to leave the yeshiva as well. A sibling who drops out of yeshiva may also provide his brother with the social support needed to make such a move.

In terms of the role played by “belonging to a newly religious family,” in the prediction of high-school dropout, poor family socialization theory may shed light on this finding. Battin-Pearson et al. (2000) used this theory as one of five selected theories to explain predictors of high-school dropout. In their study, they recognized parents’ poor resources as being a predictor of the child’s dropout. In addition, early family socialization is considered to be an influencing factor on school dropout among youth (Rumberger, 1983). Given that a newly religious family’s original socialization took place in the secular world, they must essentially start over, with no religious resources at hand, or knowledge about Ultraorthodox community’s norms. As such, for these families, “poor family socialization” may indicate their lack of religious belonging and knowledge (i.e., their “outsider-ness” in this very “insider” world), much the way poor family socialization in other contexts indicates factors such as parents’ lack of education, and parents’ divorce (Battin-Pearson et al., 2000).

In this study, neither self-esteem nor mastery was found to be a predictor of high-school dropout among Ultraorthodox Jewish youth. This finding is surprising given that a lack of personal resources in general, and lower self-esteem and mastery in particular, has been found to be characteristic of at-risk youth and connected to high-school dropout (Dillon, 2004; Aristilde, 2006; Metz, 2006). These findings may point to the unique method of education in the Ultraorthodox community; that is, to prioritize the community’s needs over the individual’s needs (Shor, 1998). When the community and family serve as such strong central controlling factors in the youths’ lives, they may become more significant than the youths’ personal resources. The reason that dropping out of high school in the Ultraorthodox Jewish community makes an even bigger statement than it does in other communities is precisely because of the priority ordinarily given by the individual to community and family sentiment, over personal sentiment.

The role of family and of community as predictors of high-school dropout was investigated via the construct of conditional regard (CR). Parental conditional positive regard was found to be a predictor of high-school dropout both (1) among dropouts vs. high school students and (2) among students in a program for dropouts vs. dropouts. These findings are consistent with previous studies that pointed to a negative contribution of PCR in regard to the well-being and loneliness of Ultraorthodox Jewish high-school dropouts (Itzhaki et al., 2018) and also with the existing literature, which presents PCPR as a practice that suppresses autonomy and leads to non-optimal self-esteem dynamics, with many negative effects such as compulsive over-investment, avoidance of challenge, and failing to develop the capacity to regulate negative emotions (Assor et al., 2004; Roth et al., 2009; Assor and Tal, 2012). Although Roth and Assor (2010) PCPR can be considered as an effective method in determining youth behavior in accordance with their parents’ expectations, it seems that for Ultraorthodox Jewish youth it can be a predictor of dropping out of high school – obviously an undesirable behavior.

Regarding PCNR, comparing high school students with dropouts, this construct made a significant contribution to group belonging both directly and indirectly via SCNR. Given the literature regarding PCNR, describing its many negative effects (Assor et al., 2004; Roth et al., 2009; Assor and Tal, 2012), the contribution of PCNR in this study is not surprising. High levels of PCNR experienced by the Ultraorthodox Jewish youngsters seemed to be a predictor of dropout. As high levels of parental closeness (closeness, satisfaction, warmth, and satisfaction with parental communication) have been found to contribute to lower odds of dropping out (Perreira et al., 2006), the lack of parental regard/love likely lessens the youth’s feeling of closeness to the parent and thus serves as a predictor of dropout.

However, comparing students in a program for dropouts vs. dropouts who were not in any type of educational framework, PCNR contributed to the group only indirectly via SCNR. As such, it appears that at some point between enrolling in an alternative educational framework and dropping out of school altogether, the parental aspect shifted to a communal aspect. This finding can perhaps be explained as follows: Yeshiva students who are at risk of dropping out are nevertheless still in the acceptable framework (they have not dropped out yet), and therefore, their “situation” is at this point known only to their parents, not to the community at large. However, when these students take the next step, leaving their sanctioned yeshivas and enrolling in alternative programs, their situation becomes “known,” as do community members’ attitudes. The community attitude, as reflected by SCNR, then becomes significant for the parents’ attitude, as reflected by PCNR.

In the current study, SCPR was not found to be a predictor of high-school dropout. According to self-determination theory (Ryan and Deci, 2000), both SCPR and SCNR would be expected to have negative consequences. However, contrary to expectations, SCPR was found in an earlier study to contribute to high levels of well-being, future orientation, and sense of community (Itzhaki et al., 2018; Itzhaki and Cnaan, 2019). It may be that participants in the current study reacted to SCPR not as a gratuitous or unwanted judgment of character, but rather as legitimate, i.e., as part and parcel of community membership. As such, it had no significant role in the dropout process. However, SCNR did have a significant role; that is, the youth clearly felt that their acceptance was predicated on giving up negatively perceived behaviors. This tension, as expressed by experiencing SCNR, was the strongest predictor of dropout in both models (i.e., high school students vs. dropouts, and students in a program for dropouts vs. dropouts). This finding is consistent with earlier studies that pointed to the negative psychological consequences of SCNR among high-school dropouts from the Ultraorthodox Jewish community (i.e., higher levels of loneliness and lower levels of well-being). The understanding that beyond the psychological harm SCNR can also serve as a predictor of dropout was a novel aspect of the current study, shedding light on the significant role played by the community in the dropout process of Ultraorthodox Jewish youth.

### Recommendations and Limitations

The unique relationship between religion and high-school dropout have been investigated before. Religious affiliation and high levels of religious involvement among mainline Protestant, conservative Protestant, Catholic, and Mormons were found to be a significant contribution in preventing high-school dropout (Lehrer, 2006; Cohen-Zada and Sander, 2008). However, there is not enough data, regarding risk and protective factors for dropout among these religious communities.

Although the present study sheds new light on risk and protective factors in explaining high-school dropout among Ultraorthodox Jewish youth, in order to generalize the current study’s finding to other religious closed communities, it would be important to investigate the role of conditional regard in other types of closed religious communities as well. Although every community has its own unique characteristic, some characteristics are common to religious communities as a whole, such as the significance of the community to its members, the importance of observing community and religious norms, and sanctions made by the community leaders and the community members when one is violating these norms (Bromley, 1991; Cates and Weber, 2013). We believe that expanding the research in this area would further our understanding of these unique characteristics, especially those related to violation of specific communities’ norms.

In terms of implementing findings, the present study points to the important role of social capital and SCR in regard to the phenomenon of high-school dropout in closed religious communities. We would recommend professionals who work with youths who are either at risk of dropping out of school or who have already done so, to focus on youths’ social capital and familiarize themselves with using conditional regard by parents and community members, and the consequences of doing so. Program interventions focusing on reducing the use of the CR practice may help in preventing youth dropout.

The current study had a few limitations. First, there were challenges in getting the agreement of the target population to participate in the research due to the closeness of the Ultraorthodox community. This challenge required our use of self-reports for the independent variables. Although self-reports are acceptable and were found reliable in other studies that have been conducted among high-school dropouts, further studies should add other forms of data, such as parents’ perception and educational success. Second, the aspect of students’ academic success/failure at school has not been examined in the current study, and it could have broadened the understanding of the high-school dropout phenomenon. We recommend that this aspect be investigated in further studies. Moreover, the study was based on cross-sectional design, which is acceptable in social sciences’ studies. However, further studies should investigate the way changes in the independent variables contribute to the dropout process. In addition, longitudinal studies may be relevant for this examination. These examinations would strength the current study’s recommendations.

## Data Availability Statement

The raw data supporting the conclusions of this article will be made available by the authors, without undue reservation, to any qualified researcher.

## Ethics Statement

The studies involving human participants were reviewed and approved by the Bar-Ilan University’s Institutional Review Board (IRB). Written informed consent to participate in this study was provided by the participants’ legal guardian/next of kin.

## Author Contributions

YI-B conceived of the study, participated in the design, data collection, analysis for the study, and drafted the manuscript. HI and YY conceived of the study, participated in its design, and contributed to drafts of the manuscript. All authors read and approved the final manuscript.

## Conflict of Interest

The authors declare that the research was conducted in the absence of any commercial or financial relationships that could be construed as a potential conflict of interest.
